# Roadkill and space use data predict vehicle-strike hotspots and mortality rates in a recovering bobcat (*Lynx rufus*) population

**DOI:** 10.1038/s41598-019-50931-5

**Published:** 2019-10-28

**Authors:** Heidi L. Bencin, Suzanne Prange, Christa Rose, Viorel D. Popescu

**Affiliations:** 1 Department of Biological Sciences, Ohio University, 107 Irvine Hall, Athens, OH 45701 USA; 2 Appalachian Wildlife Research Institute, P.O. Box, 396, Athens, OH 45701 USA; 3 Native Species Support, P.O. Box 1302, Cambridge, OH 43725 USA; 4 Sustainability Studies Theme, Ohio University, 107 Irvine Hall, Athens, OH 45701 USA; 5 Center for Environmental Research (CCMESI), University of Bucharest, 1 N. Balcescu Blvd., Bucharest, Romania

**Keywords:** Ecology, Ecological modelling

## Abstract

Roadways pose challenges for conserving wide-ranging animal species. As bobcat (*Lynx rufus*) populations recover in Ohio, an accurate evaluation of population metrics is critical to understanding future population trajectories. In this study, we integrated multiple datasets to examine overall road mortality rates in Ohio. First, we utilized a long-term vehicle-strike dataset (1978–2017) to determine landscape and local predictors of road mortality. We found that bobcats were killed at higher rates on interstates regardless of surrounding landscape composition, but that landscape variables were useful at predicting mortality on lower-traffic roads. To explore road avoidance behaviors, we used GPS telemetry data from 18 individuals to compare road crossings along trajectory paths with random road crossings simulated using Correlated Random Walks. Bobcats exhibited avoidance of certain route types (county, municipal, and US routes). Finally, by integrating traffic volume data, road crossing behavior, and accounting for the proportion of each route type present in the study area, we estimated that a minimum of 6% and up to 18% of the bobcat population in Ohio is lost to vehicle-strikes annually. To fully understand the population level impacts of this mortality, we recommend further monitoring of age structure and sex of roadkill animals. Our results identify potential areas for mitigation of vehicle-strikes and emphasize the importance of accounting for road mortality when making management decisions for Ohio’s recovering bobcat population.

## Introduction

Roads are among the most ubiquitous human features to pervade ecosystems worldwide^1,2^. The negative effects of roadways and traffic on wildlife are far-reaching; it is well known that roads can create barriers to movement^3^, cause habitat fragmentation^4,5^, impede gene flow^6^, and contribute to direct mortality^7–10^. These effects further lead to decreases in animal species abundance and richness, especially for larger-bodied mammals^11^. As human populations and infrastructure continue to expand, the commensurate increase in road density and traffic volumes bring into question the long-term viability and recovery prospects for many wildlife species^12,13^.

Vehicle strikes are a leading source of mortality for many apex predators, especially in regions with high road density, because of their large home-range requirements, extensive movements, and lack of natural predators^14,15^. Many carnivores have adapted their behavior to use roads and areas surrounding roads, as they offer least-cost movement corridors^16,17^, facilitate interspecific interactions^18^, and provide abundant food resources^19^. However, not all roads are utilized in the same way; low traffic or forest roads can be actively used for movement and foraging, whereas others, such as highways, may be actively avoided^20^. High-traffic roads can act as partial barriers to gene flow and partition animal populations into sinks and sources, thus having a direct impact on population dynamics^6,21^. As such, inferring population-level effects of road mortality is critical to understanding population trajectories, especially in unharvested populations^22^. For this, combining many types of wildlife data, such as road mortality counts, movement rates, habitat use, and genetics, along with a thorough understanding of animal space use and behavior in relation to roads, can provide better inference on population processes and validate data consistency^23^.

Bobcats were extirpated from a number of US states along the eastern coast and throughout the Midwest, including Ohio in the 1850s, as a result of heavy deforestation and over-hunting in the 19^th^ century^24,25^. In recent years, bobcat populations have begun to expand and recover in many of these regions^26,27^. As such, bobcats in the Midwest are under increasing pressure from hunting and trapping^28,29^, highlighting the need for accurate information on population dynamics. Vehicle strikes are currently considered the main source of mortality for bobcats in Ohio^30^, but the magnitude of the potential effect at the population level remains unknown. Although they exhibit sensitivity towards urbanization^31^, bobcats have been shown to cross developed areas and well-traveled roads to move between habitat fragments, causing mortality rates of up to 50% in areas with significant human development^5^, although lower rates have been recorded in other North American populations^22,32^. It is therefore critical to understand the effects of road mortality at the population level in order to determine population viability and effectively guide future bobcat management and conservation.

Focusing on the current range of bobcats throughout Ohio, the goal of this research was to evaluate the overall population effect of road mortality, and identify potential barriers to population expansion posed by roads. For this, we used a novel integration of multiple data sources and analyses to draw inference on multi-scale (local to population level) threats posed by roads to Ohio’s recovering bobcat population. Using a long-term road mortality dataset (1976–2017), we sought to determine landscape and local predictors of road mortality throughout the current bobcat range in Ohio. We then used a 3-year GPS telemetry dataset to evaluate road-crossing behavior by comparing real bobcat movements to simulated Correlated Random Walk (CRW) movements to determine whether bobcats exhibit avoidance or attraction of various route types. Lastly, we integrated information on traffic volumes and road crossing behavior while accounting for the proportion of each route type present in the study area to quantify road mortality risk at the population level. Because Ohio’s bobcat population is currently not harvested and there are no disease concerns, vehicle strikes are likely the greatest source of mortality. The information gathered from this research is therefore vital for understanding the overall population dynamics, and guiding future management and conservation strategies for this species.

## Methods

### Study area

Ohio is located near the northernmost edge of the bobcats’ native range in the midwestern US. Ohio ranks 35^th^ by geographic size (116,100 km^2^), but it is the 10^th^ most densely populated state in the US^33^. It is also home to one of the most extensive transportation systems, boasting the nation’s 5^th^ largest traffic volume, the 10^th^ largest highway network, and 4^th^ largest interstate system^34^.

Ohio contains five major ecoregions, though it is primarily composed of the Corn Belt Plains in the west and the Western Allegheny Plateau in the east. Before colonial settlement, Ohio’s land cover was up to 95% forest composed mainly of beech (*Fagus* spp.), maple (*Acer* spp.), ash (*Fraxinus* spp.), elm (*Ulmus* spp.), oak (*Quercus* spp.), hickory (*Carya* spp.), and American chestnut (*Castanea dentata*)^35^. During the 19^th^ century, most of Ohio’s original forests were cleared for human settlements, industry, and cropland, leaving the state with only 10% forest cover by the early 1900s^36^. The mid-20^th^ century saw a push for more recreational lands that brought about reforestation efforts, which shifted to include the reclamation of mining lands in the 1970s. Today, Ohio’s land cover is composed of approximately 30% forest (96.3% hardwoods, 3.7% conifers), which is primarily located throughout the Western Allegheny Plateau.

### Long-term road mortality data

**Figure 1:**
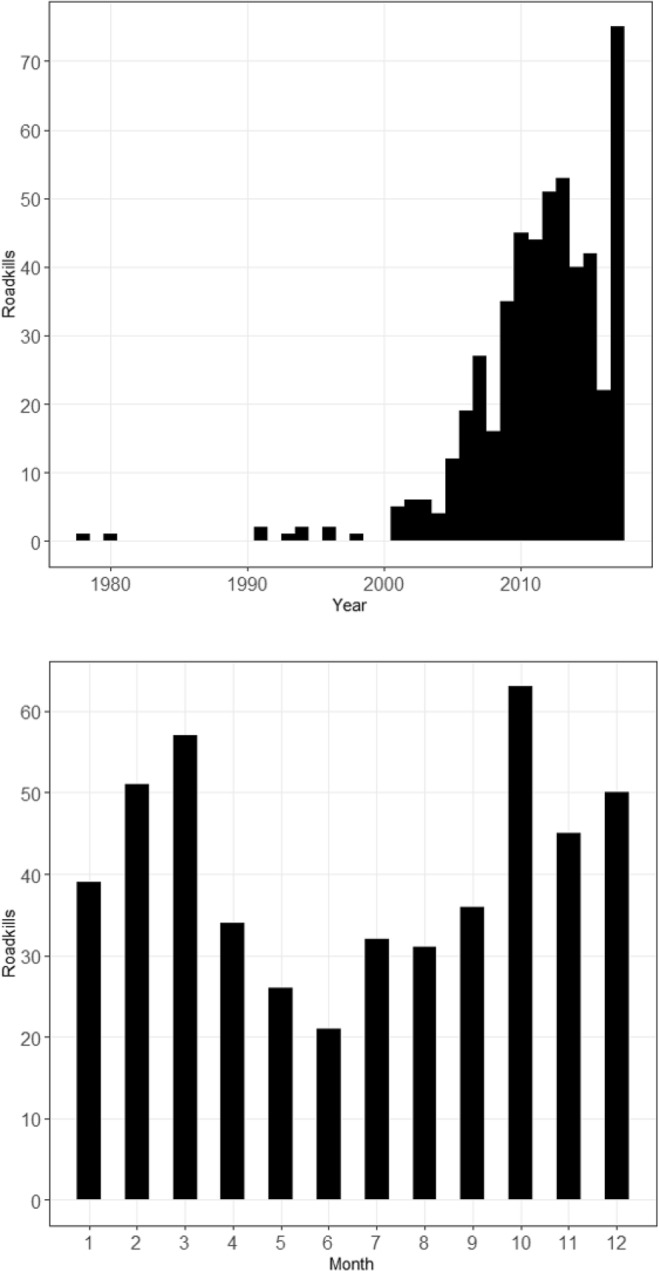
Frequency of all georeferenced Ohio bobcat roadkills (top; n = 512) per year (1978–2017), and roadkills per month across all years, excluding data from 2016 and five other incidents where month was not recorded (bottom; n = 485).

Bobcat roadkills were recorded by the Ohio Department of Natural Resources (ODNR) between the years 1978 and 2017, though only 10 incidents were recorded prior to the year 2000 (Fig. 1). Roadkills were reported by ODNR wildlife officers and personnel from other state agencies, as well as by the general public, who were encouraged to report all bobcat sightings through the yearly regulations booklet and other ODNR announcements. The original dataset contained 546 reported incidents. After eliminating entries where geospatial data were missing, 524 incidents remained. Demographic data (age and sex) were only recorded for individuals between 2006 and 2013 during a state-wide ODNR carcass collection program (Rose, unpubl. data), and therefore these variables were not used during analysis. Roadkill information only existed for part of 2016 (January-August) due to data loss.

### Road mortality analysis

To ensure that the road environment within suitable bobcat habitat was accurately represented during analysis, we only included roadkills and roads within counties that had two or more reported roadkills (33 counties, hereafter referred to as the “study area”). We mapped these roadkill occurrences (n = 512) in ArcGIS^37^ and generated four times as many random pseudo-absences (n = 2048) throughout the road mortality study area. We created pseudo-absences by generating random points within the study area in ArcGIS and snapping them to the closest road. The number of pseudo-absences was high enough to characterize the overall road network, while being distributed across the range of land cover types in Ohio^38^. To determine predictors of road mortality, we used logistic regression with a binary predictor variable (1 = roadkill and 0 = pseudo-absence) in program R^39^. We explored a suite of landscape variables (surrounding land cover and road density) and local road trait variables (route type, number of lanes) for roadkill and random points. Landscape variables were characterized as the proportion of a given land cover type within a 1000 m buffer of each roadkill and random point, whereas local variables were characterized by road traits at roadkill or random locations. We used a 1000 m buffer to represent the approximate median core area (50% fixed kernel density) of bobcats known in the region^40^. Land cover data were obtained from the 2011 National Land Cover Database (NLCD) and the most commonly occurring land cover types were grouped into three categories: forest, development, and open (including agricultural) land. We calculated road density as the length (in km) of road per 1000 m buffer. We checked for multicollinearity among land cover variables using pairwise correlations and found no highly correlated variables (>0.7 and <−0.7). We extracted road trait variables from the Ohio Department of Transportation (ODOT) spatial database for 2017 (http://www.dot.state.oh.us).

Using both land cover and road attributes, we developed a suite of 21 models and a null model. The models contained land cover variables only, road attributes only, and combinations of land cover and road attributes that we deemed biologically meaningful for predicting bobcat road mortality. We hypothesized that bobcat roadkills were positively related to the proportion of forest within a 1000 m buffer, and that roadways with higher traffic and four lanes, such as interstate highways and some US roads, also caused higher bobcat mortality. In addition to additive models, we also tested the predictive power of several interaction terms: 1) route type × forest cover; 2) route type × road density, and; 3) route type × forest cover × road density. We then used model selection procedures based on the Akaike Information Criterion corrected for small sample size (AICc) and implemented model averaging for models with a cumulative AICc weight of 0.95^41^. We calculated the odds ratios for the predictors selected in model sets based on model averaged coefficients and their unconditional standard errors. Finally, using the averaged predictions of our top selected models, we calculated the probability of mortality at each roadkill location.

### Space use data

**Figure 2:**
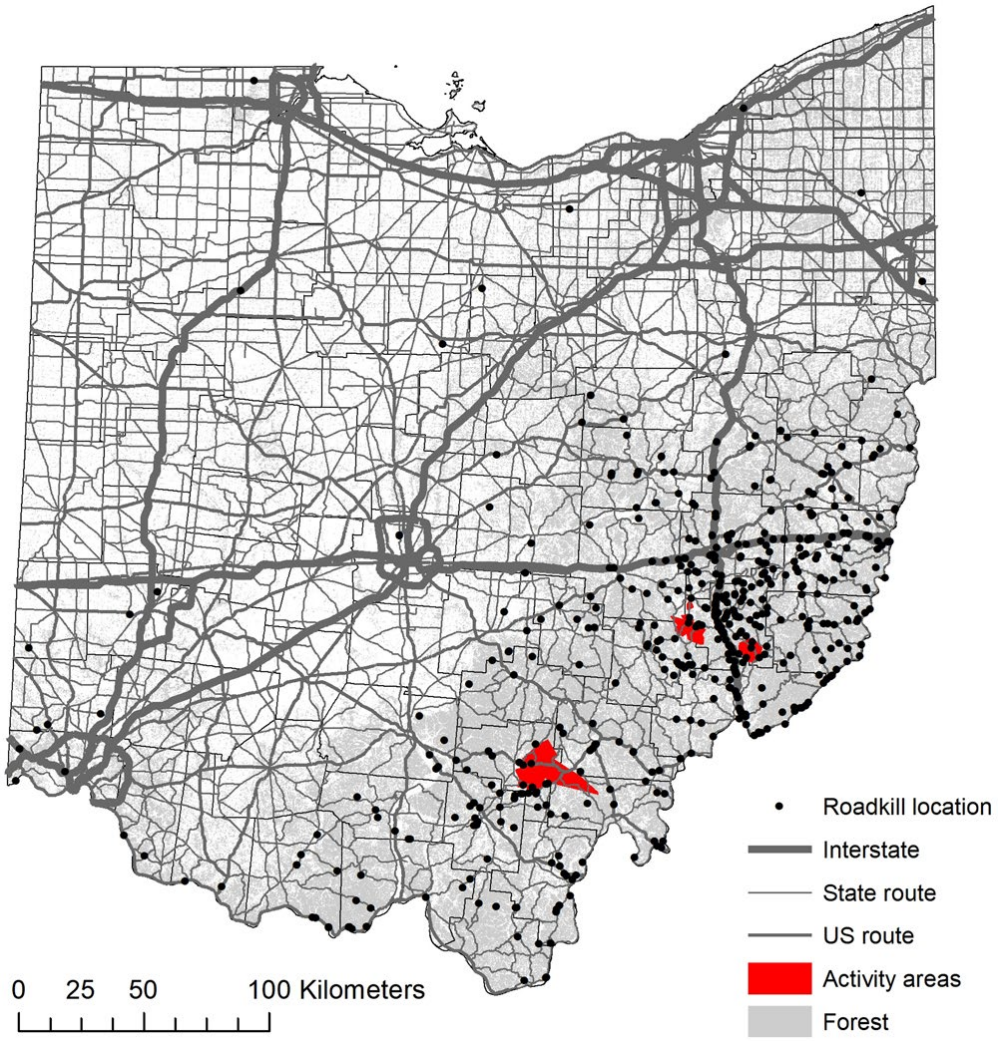
Map of Ohio showing locations of all verified roadkills, and activity areas of GPS collared bobcats (n = 18). Map generated in ArcGIS 10.4 (https://www.esri.com).

Telemetry study areas were located in the southern and eastern regions of the state, and were chosen based on the presence of two recognized subpopulations^42^ (Fig. 2). We extracted information on bobcat movements using a telemetry dataset from a study of 28 bobcats that was conducted during 2012–2015. Bobcats were fitted with Telemetry Solutions Quantum 4000 (Telemetry Solutions, San Francisco, CA) or Tellus GPS System (Followit, Lindesberg, SWE) collars that were programmed to locate individuals twice daily at 12-hour intervals on a system that rotated through a 24-hour period. GPS collars frequently located individuals only once daily, and collar performance varied; therefore, for the purposes of examining movements in relation to roads, we used a subset of data from bobcats that had GPS telemetry points recorded at an interval of 12 hours with >50 points each. Ultimately, we analyzed movement data from 18 bobcats; 10 individuals (five females; five males) in the eastern subpopulation, and eight individuals (four females; four males) in the southern subpopulation. Bobcats were either sub-adult, yearling, or adult when collared.

We used Geographic Modeling Environment (GME)^43^ to extract the movement metrics step length (linear distance between two consecutive GPS locations) and turn angle (direction of movement). We also calculated the number of road crossings by intersecting the movement paths and the roads within each bobcat’s activity area, which we defined as the 100% Minimum Convex Polygon (MCP) based only on GPS locations used in this analysis. Notably, these activity areas are not the full home ranges of individuals, as we excluded any GPS locations that were taken more than 12 hours apart, all ground and aerial telemetry locations, and all 2015 locations. We used the activity areas primarily to calculate road density metrics, evaluate bobcat road crossings, and bound the extent of random movements generated using the CRW algorithm (see next section). We did not consider age classes when evaluating activity areas.

### Ethical approval and accordance

The program administrator for Ohio’s Division of Wildlife: Wildlife Management and Research Group approved the telemetry study along with the agency’s executive administrator for wildlife research, and the wildlife federal aid coordinator (state approval codes: WFSR12 and WFPR18). Bobcat capture and handling techniques were carried out in accordance with the American Society of Mammalogists guidelines^44^. Foothold traps met criteria recommended in the Bobcat Best Management Practices^45^, but the initial trap used was replaced with a lighter one in the first season. Personnel were trained and supported by a professional USDA APHIS trapper, and were Safe-Capture International (Snohomish, WA, USA) certified.

### Bobcat behavior in relation to roads

**Figure 3:**
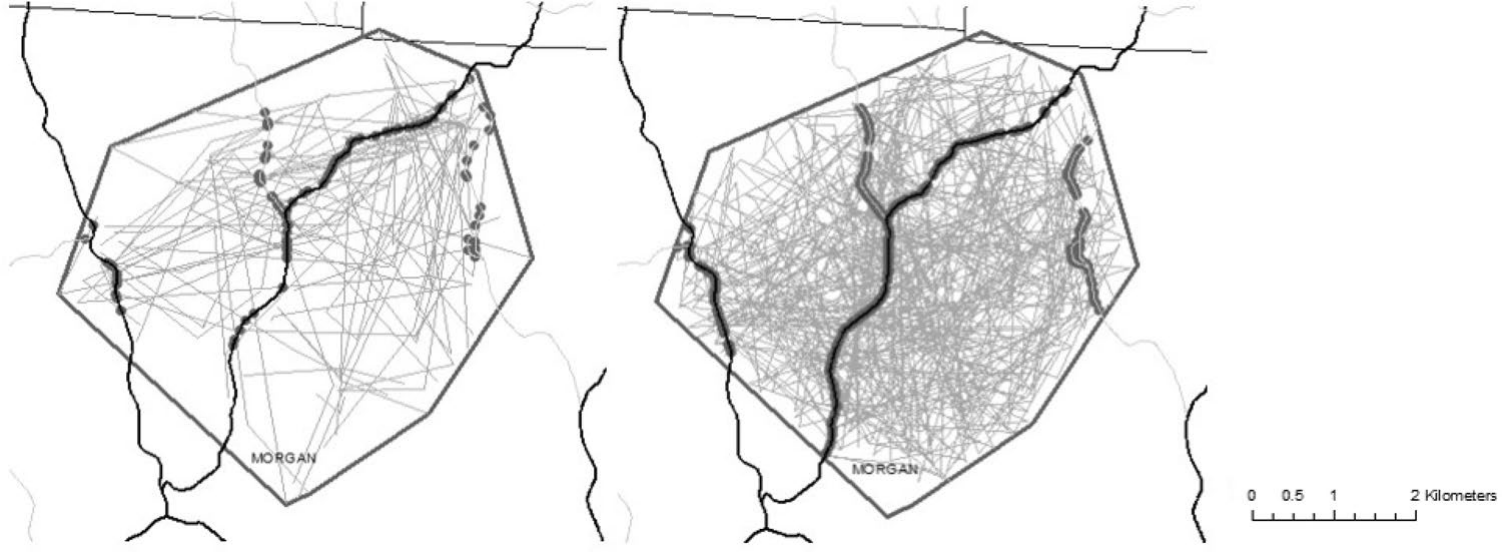
Example of projected paths (lines) and road crossings (dots) of an individual bobcat (left) and its simulated counterpart (right).

To determine if bobcats exhibited general road avoidance behavior within their activity areas, we compared inferred bobcat movements based on GPS telemetry data to random movements generated using CRWs for each individual (n = 18) in the program GME^43^. We simulated CRWs for each bobcat using the following parameters: random turn angle drawn from a uniform distribution, and distance traveled within 12 hours drawn from distributions that matched individual bobcat movement patterns. Random movements were bound by the extent of the activity areas described above. We simulated four times as many random steps compared to the real number of data points for each bobcat, to more robustly characterize potential movement across the landscape. We then extracted the intersections between the randomly generated paths and roads to create random crossings (e.g., Fig. 3).

We used a paired nonparametric Wilcoxon signed rank test to determine whether bobcats were crossing roads more or less frequently than random by comparing the number of road crossings per path (some paths crossed more than one road) between all inferred and simulated paths. To determine whether bobcats avoided certain route types at an individual level, we used a G-test of goodness-of-fit to compare the observed and expected number of crossings for each route type. The expected number of crossings assumed that the number of crossings was proportional to the different route types within each bobcat activity area. Notably, we analyzed males and females separately because male and female bobcats have different movement behaviors and space requirements, with males traveling extensively during the reproductive season and having much larger home ranges overall.

### Predicting road mortality risk at the population level

We quantified the overall mortality risk from vehicle collisions for Ohio’s bobcat population by combining space use information and road crossing behavior with traffic data. We used a road mortality risk modeling framework developed by Hels and Buchwald^46^ to estimate the probability that an individual would be killed (*P*_*killed*_) while crossing a given road. For a single road-crossing event, this can be estimated using the equation:

1 $${P}_{killed}=1-({e}^{-\frac{Na}{v}})$$

where *N* = number of vehicles/minute, *a* = width of the kill zone (m), and *v* = velocity of the animal moving through the kill zone (m/min).

For this analysis, we used metrics from a study of New Hampshire bobcats^32^; where width of the kill zone was calculated as 2.4 m and velocity of the animal moving through the kill zone was 540 m/min. Our data points, gathered 12 hours apart, precluded us from calculating road crossing speed for sampled bobcats. We used the 2016 Annual Average Daily Traffic (AADT) gathered at monitoring stations within our study area (12,859 entries) from the ODOT traffic monitoring database (https://gis.dot.state.oh.us/tims). Because bobcats are likely to be more active between the hours of 1800–0600^32^, our calculations use the average recorded traffic (24% AADT; hereafter “estimated nighttime traffic”) during this time.

To calculate the annual mortality probability (*d*_*road*_) for an individual, the annual number of road crossings can be incorporated as:

2 $${d}_{road}=1-{(1-{P}_{killed})}^{{n}_{crossings}}$$

where *n*_*crossings*_ is the number of road crossings in a given year, and *P*_*killed*_ is the probability of mortality for a single road crossing event (calculated in Eq. 1).

We calculated the mortality risk at the population level for males and females as the proportion of individuals likely to be killed on roads. We did this by considering the overall road landscape and incorporating the proportion of each route type within the counties where most reported roadkills occurred. For this, we extracted the proportion of different route types (interstate, US, state, county, township, and municipal roads) within the study area. Using AADT data, we extracted the median daily traffic value for each route type. We calculated the probability of individual mortality at one crossing (*P*_*killed*_) for each route type based on the median estimated nighttime traffic (1–15,000 cars/day). We then determined annual mortality (*d*_*road*_) of each route type based on the overall minimum, female mean, and male mean number of road crossings observed during analysis of telemetry data. To calculate the contribution of each road type to total bobcat road mortality, we conducted two further calculations. First, we multiplied the annual mortality for a given road type (*d*_*road*_) by the proportion of that road type present in our study area. Second, to account for the behavior in relation to each road type inferred from the comparisons between telemetry data and CRW simulations, we multiplied the contribution of each road type to bobcat mortality (calculated above) by the proportion of the expected mean crossings relative to township routes (the closest value to expected crossings for both sexes; see section *Bobcat behavior in relation to roads*). Because interstates were not present in bobcat activity areas, we used behavior in relation to highest traffic roads in our dataset (US routes) as a proxy. The second calculation resulted in a decrease of the contribution to overall mortality of a given road type if the road was significantly avoided, or an increase in the contribution to overall mortality if a road was used more than expected. Lastly, we calculated the annual road mortality at the population level based on the observed minimum and mean number of annual crossings, by summing the contributions of each road type to annual mortality probability adjusted for road-crossing behavior.

## Results

### Predictors of bobcat road mortality

**Figure 4:**
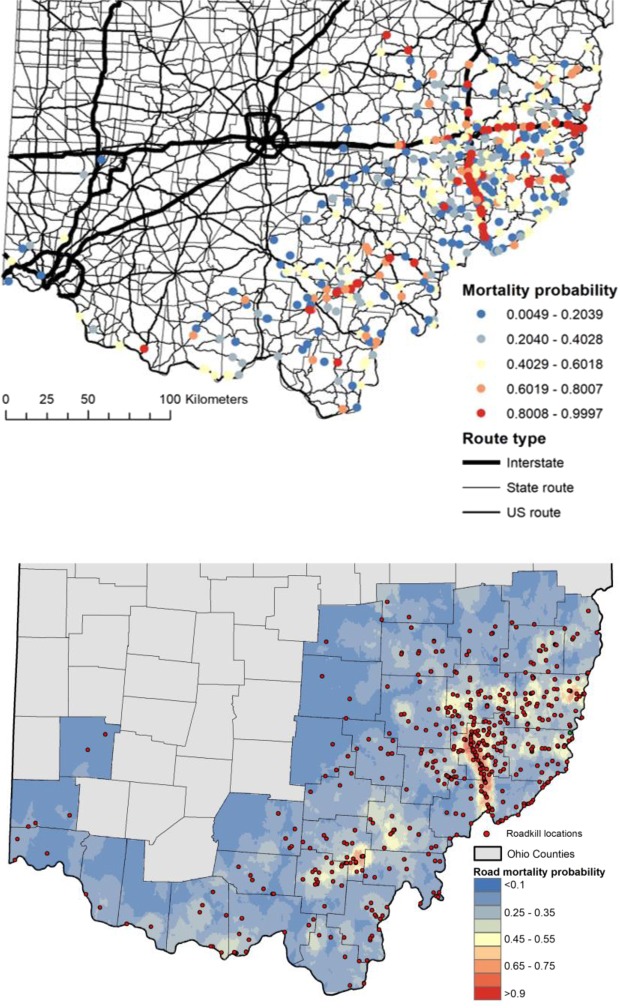
Mortality probabilities of reported bobcat roadkills based on predictors from the model average of the top three performing models (top). Spatial variation in bobcat road mortality probability based on kriging interpolation of logistic regression predictions at both roadkill and random points (bottom). Interpolation was performed using ordinary kriging based on 12 neighbors and a spherical semivariogram model in ArcGIS 10.4.

The top logistic regression model had an AUC value of 0.8576 and indicated that land cover, road traits, and the interaction term between route type and road density were important factors in predicting road mortality (Table 1). Of the 16 variables included in the top model, interstate routes were strongly associated with road mortality probability. Only two variables (% developed lands and township routes) were negatively associated with road mortality. In addition to land cover and road traits, the road density variable and the interaction term between route type and forest cover were included in the other well supported models (Table 1), indicating that road density was an important factor regardless of its interaction with route type, and that the effect of route type is dependent on the surrounding landscape. Based on logistic regression analysis, mortality probability at a given roadkill point ranged from 0.004–0.999 depending on landscape and local variables (Fig. 4).

**Table 1:** Logistic regression models describing predictors of bobcat road mortality in Ohio during the years 1978–2017.

Model Variables	K	AICc	ΔAICc	AICcWt	AUC
*NLCD* + *TR* + *RT* × *DN*	16	*1775.59*	0	*0.4*	*0.8576*
*NLCD* + *TR* + *DN* + *RT* × *FR*	16	*1776.33*	*0.74*	*0.27*	*0.8603*
*NLCD* + *TR* + *DN*	11	*1777.01*	*1.42*	*0.2*	*0.8568*
NLCD + TR + DN + RT × FR × DN	21	1777.8	2.21	0.13	0.8603
DN + TR + RT × FR	14	1795.67	20.08	0	0.8551
TR + RT × DN	13	1846.28	70.69	0	0.8462
RT × DN	12	1849.96	74.37	0	0.847
TR + DN	8	1853.47	77.88	0	0.8446
NLCD + TR + RT × FR	15	1875.3	99.71	0	0.8301
RT × FR	12	1881.08	105.49	0	0.833
TR + RT × FR	13	1882.64	107.05	0	0.8334
NLCD + TR	10	1883.45	107.86	0	0.8286
TR	7	1917.19	141.6	0	0.8145
NLCD + DN + RT × FR	10	2201.26	425.67	0	0.7538
NLCD + RT × DN	1 0	2210.68	435.09	0	0.7327
NLCD + DN	5	2233.9	458.31	0	0.7269
DN + RT × FR	8	2245.1	469.51	0	0.7456
DN + RT × DN	7	2280	504.41	0	0.6848
DN	2	2375.37	599.78	0	0.7145
NLCD + RT × FR	9	2483.38	707.79	0	0.6407
NLCD	4	2528.78	753.19	0	0.4994
Null	1	2546.96	771.37	0	0.5

Model statistics include the number of parameters per model (K), the Akaike Information Criteria score corrected for small sample size (AICc), the difference in the AICc score from the best-supported model (∆AICc), the explanatory value of each model (AICcWt), and the Area Under the Curve (AUC), denoting the predictive capability of each model. Model variables include: binned land cover types including forest, open land, and development (NLCD); road traits including the number of lanes and route type (TR); and the density of road per 1000 m buffer (DN). Interaction terms include the route type designation of township, municipal, state, US, or interstate (RT), surrounding forest cover (FR), and surrounding road density (DN). Best-supported models (those within two AICc units of the top model) are italicized.

**Figure 5:**
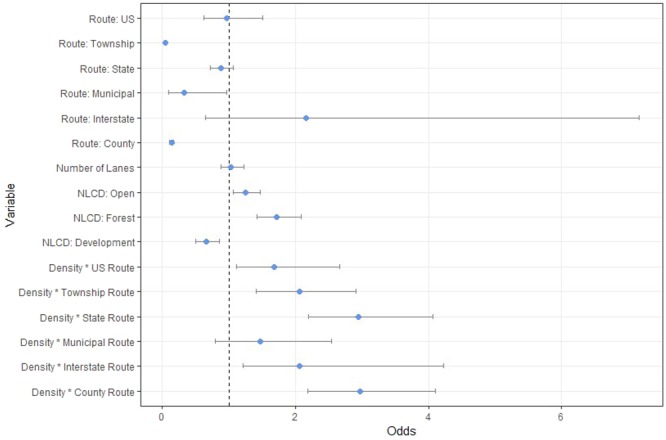
Odds ratios and confidence intervals for variables of the best-supported predictive model for Ohio bobcat road mortality. Included are road traits and land cover variables, as well as the interaction term between road density and route type. Values < 1 indicate a negative predictor of road mortality; values > 1 indicate a positive predictor of road mortality.

When evaluating the odds ratios for the 16 parameters represented in our best-supported model, all route types (except interstate routes) and one land cover type (developed land) had odds ratios < 1 (0.96–0.04), indicating a commensurate 3–95% decrease in the likelihood of a fatal vehicle strike (Fig. 5). The number of lanes was a weak positive predictor of mortality, with an additional lane resulting in a 3.2% increase in the likelihood of mortality. Roads that crossed open and forested land cover exhibited a 24% and 71% increase in the likelihood of mortality, respectively. All interaction terms were positive predictors of road mortality, ranging from a 47% (municipal route × density) to 30% (county route × density) increase in the likelihood of mortality on those route types, given an increase in the surrounding road density. Interstate routes were the only route type on its own to act as a positive predictor for road mortality, with animals being twice as likely to be killed by vehicle strikes (Fig. 5).

### Behavior in relation to roads

Cumulative time GPS collars were recording data at the resolution needed for this analysis ranged from 0.15–0.73 years (0.37 ± 0.04 years), and therefore the numbers of observed road crossings used may be underestimates of annual crossings. Minimum observed road crossings per year calculated from telemetry data were 41, with female mean number of crossings = 178, and male mean number of crossings = 195. Activity areas based on 12-hour GPS fixes varied between males and females as well as by site, though bobcat age classes were not considered. On average, females had smaller activity areas than males within the southern (female = 50 ± 12.29 km^2^; male = 115 ± 29.34 km^2^) and eastern (female = 11 ± 0.90 km^2^; male = 41 ± 9.04 km^2^) populations. Three individuals (two used in our analysis) were potentially killed by vehicle-strikes on roads^40^.

Density of roads within activity areas ranged from 0.6–1.5 km/km^2^ and was similar between males (south = 1.2 ± 0.09 km/km^2^; east = 0.94 ± 0.15 km/km^2^) and females (south = 0.94 ± 0.07 km/km^2^; east = 1.0 ± 0.20 km/km^2^). Road crossings per path (calculated as crossings per path between two successive GPS locations taken 12 hours apart) were greater for males (south = 1.14 ± 0.34; east = 0.75 ± 0.13) than females (south = 0.47 ± 0.07; east = 0.66 ± 0.08), though the total average number of paths within the needed resolution for analysis were fewer for males (south = 211, east = 192) than females (south = 335, east = 308). The number of road crossings per path was not significantly different between real bobcat movements (0.749 ± 0.10 road crossings/path) and simulated movements (0.917 ± 0.12 road crossings/path) (V = 47, Wilcoxon test p = 0.0987).

When investigating whether individuals were avoiding or favoring crossing certain route types (Table 2; except interstate routes), 67% of individuals (n = 12; 7 females and 5 males) exhibited road-crossing behaviors significantly different than would be expected based on the proportion of route types available within their activity area. For bobcats where the G-test was significant, similarities and differences existed between female and male behavior (Table 3). Given that a route type was present in an individual’s activity area, more bobcats had fewer than expected crossings, denoting road avoidance of three route types: municipal, county, and US routes. Females showed greater avoidance of county roads (mean observed/expected crossings = 0.66) than males (0.95), whereas males exhibited a greater avoidance of US routes (males = 0.33; females = 0.91). Males crossed state routes marginally less than expected (0.96), whereas females crossed state routes more than expected (1.11). Both males and females crossed township routes at rates that were near expected (male = 1.01; females = 1.06). However, not all route types were present in all activity areas for this subset; county roads were only present in 71% of female activity areas, municipal roads were present in 14% of female and 40% of male activity areas, and US routes were present in 29% of female and 40% of male activity areas (Table 3). No interstate roads were present within telemetry study areas.

**Table 2:** G-test goodness-of-fit output for the observed vs. expected number of road crossings an individual bobcat made, given the proportion of route types available in its activity area.

Bobcat ID	Observed Road Crossings					Expected Road Crossings (df = 4)						p-value (2-tailed)
	CR	MR	SR	TR	US	CR	MR	SR	TR	US	G	
4	**6**	**0**	**2**	**105**	**10**	**17.39**	**0.00**	**11.18**	**77.03**	**17.39**	**34.32**	**0.0000**
5	**86**	**0**	**16**	**164**	**2**	**57.65**	**7.18**	**20.87**	**166.49**	**15.80**	**47.07**	**0.0000**
**6**	57	0	18	158	0	56.49	0.00	27.99	148.53	0.00	4.68	0.3220
**7**	9	0	4	28	1	10.34	0.00	7.62	20.81	3.24	6.63	0.1570
8	**149**	**0**	**0**	**160**	**0**	**97.01**	**0.00**	**51.40**	**160.58**	**0.00**	**126.71**	**0.0000**
10	**0**	**0**	**64**	**118**	**0**	**0.00**	**0.00**	**32.93**	**149.07**	**0.00**	**29.90**	**0.0000**
**11**	62	0	66	9	0	50.55	0.00	74.28	12.18	0.00	4.29	0.3680
12	**26**	**0**	**54**	**32**	**14**	**26.67**	**3.40**	**14.10**	**70.59**	**11.24**	**99.21**	**0.0000**
14	**6**	**0**	**10**	**167**	**0**	**13.68**	**0.00**	**23.63**	**145.68**	**0.00**	**18.52**	**0.0010**
15	**0**	**0**	**49**	**202**	**0**	**0.00**	**0.00**	**104.37**	**146.63**	**0.00**	**55.33**	**0.0000**
**16**	35	0	114	32	0	43.97	0.00	96.10	40.92	0.00	7.22	0.1250
17	**129**	**7**	**62**	**227**	**15**	**87.01**	**13.84**	**58.93**	**251.96**	**28.26**	**31.99**	**0.0000**
18	**22**	**0**	**2**	**250**	**0**	**34.25**	**0.00**	**12.94**	**226.81**	**0.00**	**21.73**	**0.0002**
19	**40**	**0**	**112**	**51**	**0**	**60.18**	**0.00**	**49.43**	**93.40**	**0.00**	**88.85**	**0.0000**
**20**	1	0	99	68	0	4.89	0.00	109.46	53.65	0.00	9.17	0.0569
**21**	31	0	82	4	0	41.87	0.00	69.49	5.64	0.00	5.76	0.2170
22	**5**	**0**	**6**	**30**	**0**	**11.00**	**0.00**	**10.48**	**19.53**	**0.00**	**11.19**	**0.0245**
23	**0**	**0**	**39**	**37**	**0**	**5.46**	**0.00**	**41.23**	**29.31**	**0.00**	**12.91**	**0.0117**

Individuals where the expected outcome was significantly different from the observed are denoted in bold (n = 12). Road types include: county (CR), municipal (MR), state (SR), township (TR) and U.S. routes (US).

**Table 3:** Mean proportions of observed/expected values for female and male bobcats that showed a significant difference from expected road crossing behaviors (n = 12; 7 females, 5 males).

	Township	Municipal	County	State	US
Females	1.0558	0.0000	0.6589	1.1130	0.9103
% females	100%	14%	71%	100%	29%
Males	1.0141	0.2529	0.9472	0.9623	0.3287
% males	100%	40%	100%	100%	40%

Values <1 indicate avoidance of a route type. Values >1 indicate that individuals crossed a route type more than what would be expected, given the proportion of that route type available in their activity area. The percent of individuals with a given route type present in their activity areas is also listed.

### Mortality risk at the population level

**Figure 6:**
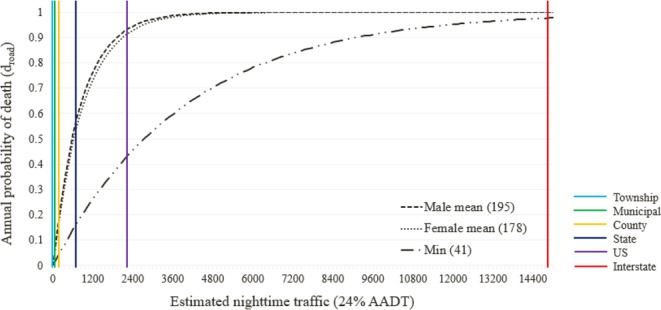
Annual probability of Ohio bobcat death (*d*_*road*_, Eq. 2) based on traffic and the minimum, female mean, and male mean observed road crossings per year. The width of the kill zone (*a*) = 2.4 m, and velocity of the animal moving through the kill zone (*v*) = 540 m/min. The estimated nighttime traffic values for each route type are marked.

The likelihood that a bobcat would be killed on roads during a single crossing ranged between 0.01% for low traffic roads (estimated nighttime traffic = 24 vehicles) and 8.84% for high traffic roads (estimated nighttime traffic = 15,000 vehicles). The annual mortality probability at various estimated nighttime traffic values was also calculated based on the minimum (41), female mean (178), and male mean (195) road crossings observed by bobcats through telemetry data (Fig. 6). However, these annual probabilities assume that animals have the same propensity of crossing high-traffic roads and low traffic roads.

The proportion of route types within the study area varied from 0.02 (interstate routes) to 0.42 (township routes). The probability of mortality for a single road-crossing event (*P*_*killed*_) was between 0.0002–0.088, depending on the traffic levels. Annual mortality probability (*d*_*road*_) was between 0.0096–1 depending on route type (Table 4). The contribution of each route type to annual mortality at the population level depended on both the proportion of each road type in the study area, and the behavior of bobcats in relation to each road type (section Behavior in relation to roads). The contribution of each road type ranged between 0 (municipal roads) and 0.08 for state routes (or 8% for the mean number of 178 crossings for females), with lower levels of mortality under the scenario considering a minimum of 41 crossings per year (Table 4). These contributions resulted in cumulative probabilities of annual road mortality risk at the population level of 0.1803 (mean female crossings scenario), 0.1789 (mean male crossings scenario), and 0.0604 (minimum crossings scenario), suggesting a minimum estimated population loss from vehicle strikes of 6% annually, and a mean of 18%.

**Table 4:** Annual, weighted, and cumulative weighted mortality probabilities based on the proportion of each route type and its corresponding estimated nighttime traffic value (24% median AADT) available within current bobcat range (33 counties), as well as demonstrated behavior in relation to each route type. Displayed for three possible annual crossing values: mean female (178), mean male (195), and minimum (41) inferred crossings.

Route type	% Road	24% median AADT	*P*_*killed*_	Annual mortality probability (*d*_*road*_) at n crossings			Annual mortality (*d*_*road*_) weighted for road proportions			Annual mortality (*d*_*road*_) weighted for road proportions and behavior		
				Mean female	Mean male	Min	Mean female	Mean male	Min	Mean female	Mean male	Min
Township	0.4183	38	0.0002	0.0409	0.0447	0.0096	0.0171	0.0187	0.004	0.0171	0.0187	0.004
Municipal	0.1074	88	0.0005	0.0922	0.1005	0.022	0.0099	0.0108	0.0024	0	0.0028	0.0017
County	0.2871	233	0.0014	0.2259	0.2446	0.0573	0.0649	0.0702	0.0164	0.0379	0.0675	0.013
State	0.1321	785	0.0048	0.5779	0.6113	0.1802	0.0763	0.0807	0.0238	0.0799	0.0728	0.0232
US	0.0378	2346	0.0144	0.9241	0.9406	0.4477	0.0349	0.0355	0.0169	0.0303	0.0115	0.0092
Interstate	0.0173	14931	0.088	1	1	0.9772	0.0173	0.0173	0.0169	0.015	0.0056	0.0092
					**Cumulative weighted probabilities**		0.2204	0.2333	0.0804	**0.1803**	**0.1789**	**0.0604**

## Discussion

Our study revealed that the overall impacts of roads on Ohio’s recovering bobcat population, based on its current distribution, is high. This is particularly important given the species’ recent reestablishment in the state, and the fact that Ohio has one of the highest road densities and traffic volumes in the US^34^. This study supports the hypothesis that bobcat roadkills are positively related to certain surrounding landscape variables, and that roadways with higher traffic and more lanes also cause higher bobcat mortality. Specifically, we showed that (1) bobcats are being killed at higher rates on interstates regardless of surrounding landscape variables, but on smaller roads the probability of death is more dependent on surrounding landscape variables; (2) bobcats cross roads at a rate not significantly different than would be expected overall, but they do show avoidance of certain route types, with some differences between males and females, and; 3) weighted mortality probabilities that combine the relative proportion of route types in the study area with bobcat behavior indicate a minimum estimated population loss from vehicle strikes of 6% annually, and a mean of 18%. However, the population-level impacts of estimated annual road mortality are highly dependent on the age and sex structure of the segment of the population killed by vehicle strikes, which warrants further research.

### Predictors of road mortality

Odds ratio results from our logistic regression analysis of landscape and local predictors of road mortality, based on a 40-year dataset, show clear positive and negative predictors of mortality that often relate to results gathered during behavioral analysis of telemetry data. Township, municipal, and county routes were strong negative predictors of road mortality. Interestingly, township routes were crossed at nearly the expected rate by both males and females sampled from the telemetry study, given the proportion of routes available within individual activity areas. This indicates that bobcats are frequently crossing township routes but rarely suffer vehicle-strikes on them. Municipal routes are focused around areas of intense urbanization, which bobcats tend to avoid^5,31^. In fact, very few bobcats from the telemetry study sample had municipal routes in their activity areas, and all of those that did crossed less than expected. Bobcats also tended to avoid county routes (females more so than males). Overall this suggests that township, municipal, and county routes, especially in areas of low road density and forest cover, pose little threat to bobcats due to either low evidence of road mortality or avoidance behavior; our analysis of the GPS telemetry dataset supports this assertion.

US and state routes were less likely to facilitate mortality, though they both had upper confidence intervals > 1, suggesting they could act as weak positive predictors (Fig. 5). Interstate routes are often associated with increased mortality^32,47,48^, and were the only route type to act as a strong predictor of road mortality, regardless of the surrounding road density. Number of lanes had an odds ratio >1, which falls in line with the results for different route types (township, municipal, and county routes being not predictive, state and US routes being somewhat predictive, and interstate routes having strong predictive value).

Of the three major land cover types tested, open land and forest were positive predictors (Fig. 5). Road density was included in the top two models, suggesting that an overall denser road network resulted in higher roadkill. This supports the findings of similar studies^32^, and suggests that surrounding road density is an important factor in the overall impacts of road mortality. At the same time, land cover variables were included as an interaction term with route type in the second best model, suggesting that higher percent forest within the immediate vicinity of any road results in higher mortality. These findings corroborate a study in Texas, where bobcats were more likely to be killed in vehicle strikes on stretches of road adjacent to preferred habitat, or which contained preferred habitat in the median^47^. The negative association detected between developed lands and roadkill also corroborates our finding that areas with municipal roads (i.e., urban and suburban areas) were rarely utilized by bobcats.

### Behavior in relation to roads

Overall, we found that bobcats made fewer than expected road crossings among the various route types, particularly municipal, county, and US routes. However, avoidance was often marginal (county and state routes for males; US routes for females) and differed between sexes. Notably, females demonstrated greater than expected road crossings for state routes, whereas males did not. Instances of marginal or no observed road avoidance behavior relates to findings from other studies. Dickson and Beier^49^ found that cougar home ranges were not typically located near high-speed roads, but that individuals did not avoid areas surrounding roads within their home range, especially when they were located in preferred habitat. However, the lack of interstates in bobcat activity areas was likely an artifact of study site location.

Behavior in relation to roads also depends on where an individual’s core area is located within its broader home range, which may be influenced by resource availability, territories of neighboring individuals, and connectivity^50^. Avoidance of larger roads may be the result of perceived danger^32^. The findings on behavior in relation to roads are subject to the limitations imposed by the GPS data that recorded bobcat movement at a 12-hour interval. This temporal resolution was too coarse for evaluating fine-scale movement behaviors, which influenced our ability to pinpoint road crossings with greater accuracy and determine the absolute number of road crossings.

### Mortality risk at the population level

Our estimates of annual road mortality at the population level, when accounting for proportions of route types present and number of road crossings, range from minimum of 6% to a mean of 18% for both females and males - an estimate that is similar to those predicted for other large carnivores^11^. It is not uncommon for carnivores to have high mortality attributed to roads in areas of increased traffic volume or road density. For example, Kaczensky *et al*.^48^ found that, in a region containing a high traffic volume, 31% of known brown bear (*Ursus arctos*) mortality was due to vehicle strikes over a seven year period; documented Florida panther (*Puma concolor coryi*) deaths consisted of 49% road mortalities^51^, and; an increase in the number of roads was suggested to be the most likely cause of a 30% decrease in a badger (*Meles meles*) population over 20 years^52^. Felid-focused studies found both higher and lower mortality estimates compared to ours, and they were context dependent. Dickson and Beier^49^ reported 32% of radio-collared cougars were killed by vehicles in southern California, another road-dense region. In areas of low road density such as Texas, where road densities are less than half those present in Ohio (1.56 km of lane/km^2^ in Texas compared to 3.64 km of lane/km^2^ in Ohio)^53^, bobcat and ocelot (*Leopardus pardalis*) populations are strongly impacted by highways^47,54^. In another region with low road density, New Hampshire, bobcat annual road mortality was reported to affect only 1.4% of the population. Although we estimated an annual mortality in our study area of 6–18%, these estimates are subject to variation induced by spatial differences in abundance, and the overall impact on the population strongly depends on the sex and age of roadkill animals (see next section). This estimate is supported by the proportion of radio-collared bobcat fatalities potentially related to vehicle-strike during the telemetry study (10.7% of study animals, though the fates of all animals were not known), and by a study of another recovering bobcat population in Illinois, where vehicle-related mortality was approximately 10%^22^. It should be noted that seasonality, land cover, and surrounding road density were not factored into these rough estimates of population-level mortality rates, though doing so would strengthen the predictive power of these estimates, particularly given our findings from the analyses of long-term road mortality data.

### Differences between two subpopulations

Although our results provide important insight into the overall mortality caused by roads in Ohio, further research is needed to explain apparent and observed differences between the two Ohio subpopulations, which are thought to require different management strategies^24,42^, with more conservative measures taken for the southern population. Previously, Anderson *et al*.^42^ identified genetic distinctions between the two populations and suggested the potential for a sink population in the south. The radio-collared individuals we used for analysis had broader areas of movement in the southern population, though age classes were not considered during analyses. Although it has been shown that the two subpopulations have no distinguishable differences in overall diet, it has been suggested that there may be poorer resource availability in the south^24^. Currently, the magnitude of these differences and their driving forces are unknown.

Poor resource availability has been associated with high adult mortality and little or no kitten recruitment, to which felid populations are notably sensitive^55,56^. When combined with road mortality, poor resource availability could have cumulative effects. This is especially relevant given the seasonal variation apparent in vehicle strikes (Fig. 1) that show peaks during the mating (February–April) and late kitten rearing (October) seasons in Ohio, when males are roaming farther distances and juveniles are leaving their natal ranges, respectively. Because the relative proportions of adult females and juveniles killed on roads impacts the number of breeding individuals and those making it to reproductive age, respectively, these factors have the potential to influence the trajectory of the population. While there is evidence that more younger animals and more males were killed on Ohio roads between 2006 and 2013 (Rose and Prange, unpubl. data), continuing to monitor both the age and sex structure of animals killed on roads is critical for understanding the potential of road mortality to affect population viability. Though we were unable to do so, incorporating demography into the model would allow for improved inferences on which life stage and sex are being impacted the greatest by roads.

### Implications for management and conservation

Mitigation of wildlife-vehicle strikes can be a critical component of management and conservation strategies for populations where road mortality is high. Roughly 49% of reported bobcat roadkills in Ohio occurred on state routes, though given their ubiquity throughout the landscape, they are proportionally not as likely to result in mortality as interstate routes. Given these results, we recommend mitigation measures to increase habitat connectivity, as this may help ameliorate the effects of habitat fragmentation caused by roadways^57,58^. These measures may be useful not only on interstate routes, but also other high-traffic roads in areas of significant surrounding forest, with particular focus on potential wildlife corridors. One of the most common approaches towards increasing connectivity on large roadways are culverts or underpasses, which are most effective when properly sized and positioned in the landscape^8^. In areas of high mortality risk where adding a culvert is not feasible, roadway signs alerting of potential animal crossings are a simple and economical measure to increase driver awareness, and may be particularly effective for charismatic species such as bobcats.

Ohio is one of the most road-dense states in the US, with suitable bobcat habitat that is restricted primarily to the south and east, and highly fragmented. Similar to other carnivore species, bobcats have large home-range requirements and low reproductive rates, making the threat posed by roads potentially great^47^. While a harvest rate of 20% is typically assumed as sustainable for established populations^56^, this may be confounded by environmental factors^59^. We recommend these considerations be factored into any future management plans for bobcats; it is likely that the higher road density present in the state is leading to higher baseline rates of mortality. Particular attention needs to be given to the eastern population, where two major interstate highways (I-70 and I-77) pose a disproportionately larger threat. We also suggest the completion of a Population Viability Analysis (PVA) to determine baseline population statistics, predict the population trajectory for the two Ohio bobcat subpopulations, and help identify management strategies that will allow for the population to persist in the long term. 

## Supplementary information

Supplementary Information

## Data Availability

The traffic data used during the current study are available in the ODOT repository, [https://gis.dot.state.oh.us/tims]. Additional datasets are available from the corresponding author on reasonable request.
