# Author Correction: Roadkill and space use data predict vehicle-strike hotspots and mortality rates in a recovering bobcat (*Lynx rufus*) population

**DOI:** 10.1038/s41598-020-62929-5

**Published:** 2020-04-03

**Authors:** Heidi L. Bencin, Suzanne Prange, Christa Rose, Viorel D. Popescu

**Affiliations:** 1 Department of Biological Sciences, Ohio University, 107 Irvine Hall, Athens, OH 45701 USA; 2 Appalachian Wildlife Research Institute, P.O. Box, 396, Athens, OH 45701 USA; 3 Native Species Support, P.O. Box 1302, Cambridge, OH 43725 USA; 4 Sustainability Studies Theme, Ohio University, 107 Irvine Hall, Athens, OH 45701 USA; 5 Center for Environmental Research (CCMESI), University of Bucharest, 1 N. Balcescu Blvd., Bucharest, Romania

Correction to: *Scientific Reports* 10.1038/s41598-019-50931-5, published online 28 October 2019

In this Article, there is an error in the ‘Long-term road mortality data’ subheading in the Methods:

“Demographic data (age and sex) were only recorded for individuals between 2006 and 2013 during a state-wide ODNR carcass collection program (Rose, unpubl. data)” - should refer to an Article in press – Reference 1 in this Correction - rather than unpublished data.
