# Supplementary material for: Roadkill and space use data predict vehicle-strike hotspots and mortality rates in a recovering bobcat (Lynx rufus) population
Source: Sci Rep. 2019 Oct 28;9:15391. doi: 10.1038/s41598-019-50931-5 (PMC6817856; doi:10.1038/s41598-019-50931-5)
Supplement: Supplementary file 1 — Supplementary Information [file 41598_2019_50931_MOESM1_ESM.pdf]

Supplementary Information

Manuscript title: Roadkill and space use data predict vehicle-strike hotspots and mortality rates in a recovering bobcat (Lynx rufus) population

Authors: Heidi Bencin, Suzanne Prange, Christa Rose, Viorel Popescu

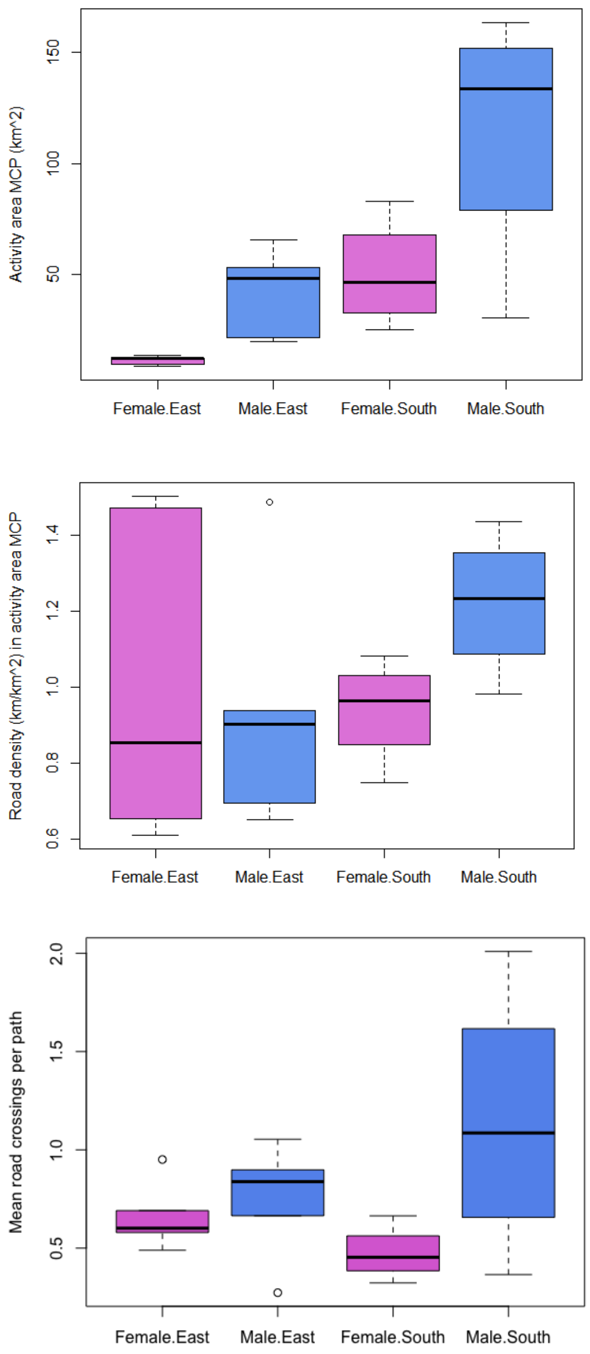

Medians and interquartile ranges of Ohio bobcat activity areas (top), road density (center), and mean road crossings per path (bottom) based on gender and subpopulation ($n = 18$; $n_{female,east} = 5$, $n_{male.east} = 5$, $n_{female.south} = 4$, $n_{male.south} = 4$). Age class of bobcats was not considered.

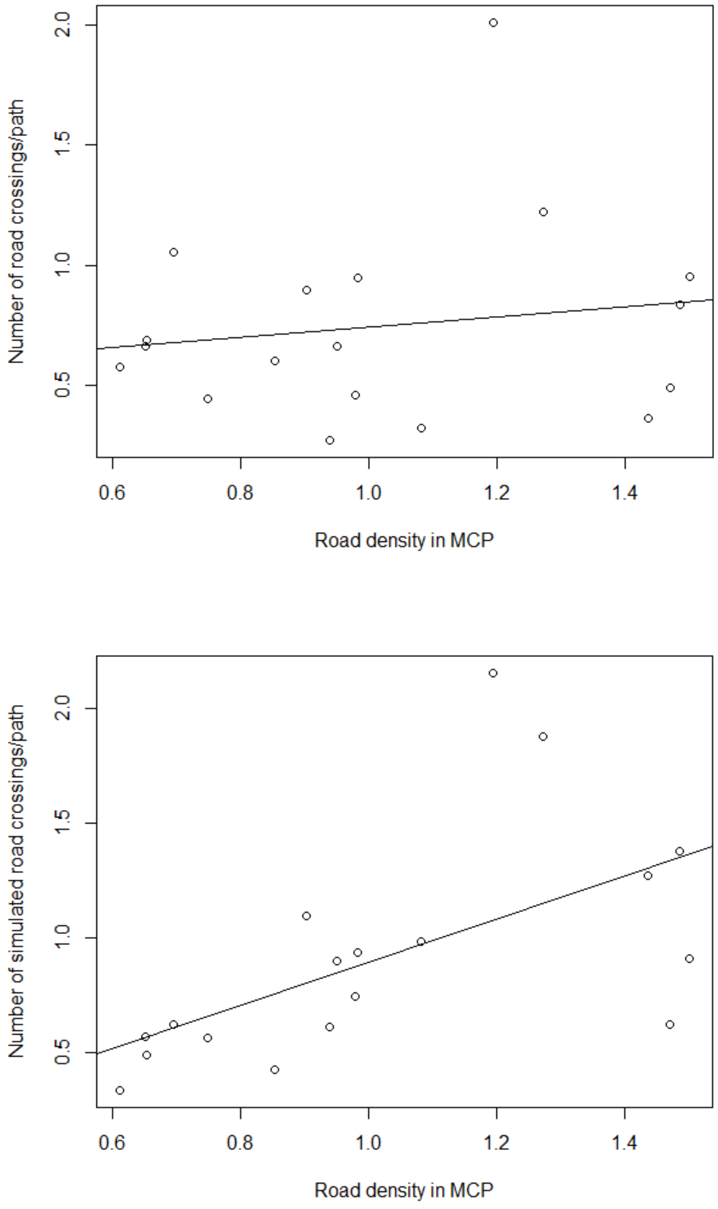

The number of road crossings per path as a function of road density within an individual Ohio bobcat 's activity area (MCP) is not significantly different (V = 47, Wilcoxon test p = 0.0987) between real bobcat paths (top) and simulated paths (bottom).
